# A Novel *in vivo* System to Test Bronchodilators

**DOI:** 10.16966/2470-3176.120

**Published:** 2017-02-03

**Authors:** Kenneth J Addison, John Morse, Annette Robichaud, Michael O Daines, Julie G Ledford

**Affiliations:** 1 Department of Medicine, University of Arizona, Tucson, Arizona, USA; 2 SCIREQ Inc., Montreal, Québec, Canada; 3 Pediatrics, University of Arizona, Tucson, Arizona, USA; 4 Immunobiology, University of Arizona, Tucson, Arizona, USA

**Keywords:** Bronchodilators, *in vivo*, Asthma

## Abstract

The incidence and severity of asthma continue to rise worldwide. β-agonists are the most commonly prescribed therapeutic for asthma management but have less efficacy for some subsets of asthmatic patients and there are concerns surrounding the side effects from their long-term persistent use. The demand to develop novel asthma therapeutics highlights the need for a standardized approach to effectively screen and test potential bronchoprotective compounds using relevant *in vivo* animal models. Here we describe a validated method of testing potential therapeutic compounds for their fast-acting efficacy during the midst of an induced bronchoconstriction in a house dust mite challenged animal model.

## Introduction

Asthma, a chronic and often long-term lung disease, is characterized by inflammation and reversible airway obstruction. The incidence and severity of asthma continue to rise worldwide and as many as 24 million Americans (7.4% of adults and 8.6% of children) were reported to have asthma in 2015 [1]. Asthma affects people of both genders and is independent of age, socio-economic background and racial ethnicity. While most asthma patients are well-managed with proper physician-guided treatment and preventative measures, there is no cure and subsets of asthmatics respond poorly to common therapies. A significant cause of morbidity and mortality in asthma involves acute exacerbations, which can lead to airway injury, remodeling, lung function decline and even death [2–4]. Exacerbations in severe asthmatics are particularly concerning, as exacerbations in this population are associated with accelerated lung function decline [2].

Since reduced lung function is a risk factor for severe exacerbation [5,6], this vicious cycle promotes an exacerbation-prone phenotype of asthma. In addition, there are concerns surrounding the side effects from long-term use of the most frequently prescribed asthma medications: corticosteroids [7] or short acting β2-agonists [8,9]. Therefore, the rise in asthma prevalence, the need for better control of exacerbations and the desire to reduce long-term side effects of commonly used asthma drugs, has elevated the need for a standardized approach to effectively screen and test potential bronchoprotective compounds using relevant *in vivo* animal models.

Currently, the forced oscillation technique (FOT) is the gold standard to perform pulmonary function tests (PFTs) in small animal *in vivo* studies [10,11]. This technique provides accurate and reproducible lung function measurements of animal subjects by applying a brief, small amplitude, oscillated airflow test signal at the subject’s airway opening and characterizing the relationship between the input and output signals [10].

A single sinusoidal wave of predefined frequency (2.5 Hz) is used in a single compartment model of lung mechanics to allow for measurements to determine the overall resistance (Rrs) and elastance (Ers) of the entire respiratory system [12]. In contrast, broadband low frequency measurements are conducted using a range of mutually prime frequency waves covering a broad spectrum (1–20.5 Hz). These measurements are used in the constant phase model to dissect out values associated with the central (Rn) and peripheral airways (G, H) [12].

While the technique can be adapted for a variety of animal models, the mouse is by far the most commonly used species in asthma research. As described by Bates & Irvin [13], the technique requires that the subject be anesthetized, tracheotomized or orally intubated, and mechanically ventilated. Automated sequences of PFT measurements can be predefined, using scripts, to reproducibly capture the respiratory systems overall changes in obstruction and stiffness and/or the partitioning between central airway and peripheral tissue mechanics. PFTs can be performed under baseline conditions as well as through a series of methacholine challenges to assess airway responsiveness in test animals [11].

While researchers have adopted various methods to model the use of bronchodilator compounds *in vivo,* their delivery methods (subcutaneous infusion, intraperitoneal injections, intra-tracheal instillations) have not always been consistent with the clinical delivery of the bronchodilator agent [14,15]. Recent studies have used administration techniques more consistent with drug delivery in a clinical setting, using either a nebulizer, a nose-only inhalation, or an intranasal delivery technique [16–22]. While information was gained in all of the aforementioned studies, consistent validated methods testing for potential novel therapeutic inhaled compounds with fast-acting immediate relief from rapid onset bronchoprotective effects during a bronchoconstrictive event have yet to be published.

We hereby describe a modified experimental approach to test for potential fast acting bronchoprotective compounds during a methacholine challenge. We validated our approach using a common asthma bronchodilator, Albuterol Sulfate (Nephron Pharmaceuticals Corporation), a fast-acting inhaled short acting β2-agonist used for the rapid reversal of bronchoconstriction in asthmatic patients.

## Methods

**Figure 1:**
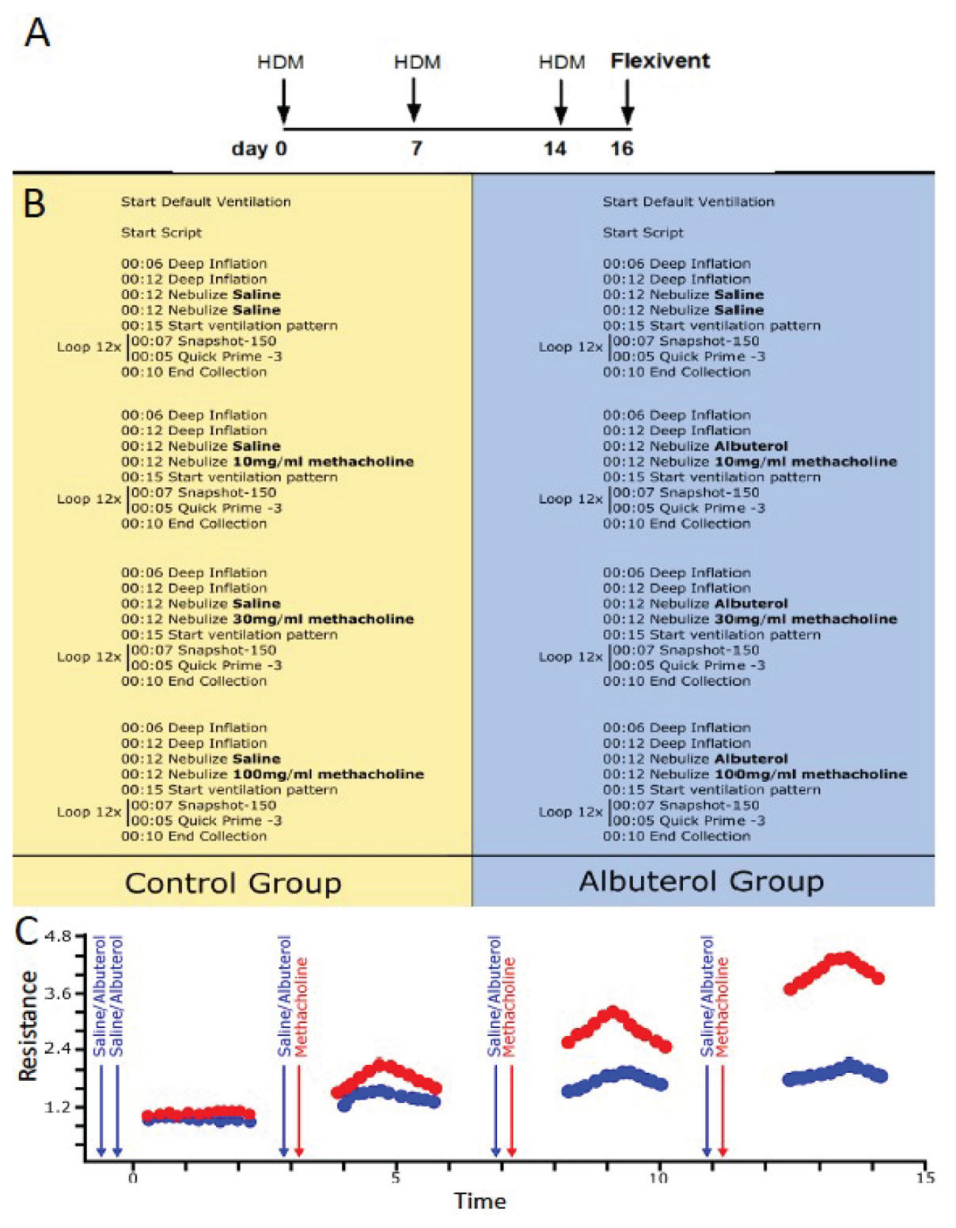
**A**) Mice were challenged with HDM on days 0, 7 and 14. On day 16, all HDM challenged mice were divided into two groups **B**): Control group (left panel); nebulized with saline, and Albuterol group (right panel); nebulized with Albuterol Sulfate (0.083% in saline). **C**) Immediately after, both groups received an aerosolized methacholine bronchoconstrictor challenge (10, 30, 100 mg/ml) and lung function was recorded using the forced oscillation technique. Arrows indicated when saline or albuterol and methacholine were administered.

In order to validate our method, we used the common allergic airway model in which 8–10 week old Balb/C male and female mice that were sensitized and challenged with 100 μg house-dust mite extract (HDM; prepared from dry weight, Greer) over the course of three intranasal instillations while under isoflurane anesthesia (Figure 1A). Two days after the last HDM challenge, mice were anesthetized with urethane (Sigma, U2500) prepared at a concentration of 125 mg/ml in sterile dH_2_O and dosed at 16 ul/gram of body weight intraperitoneally. Once a surgical plane of anesthesia was reached, the subject’s trachea was cannulated with a 19-gauge metal cannula using previously described methods [11,23]. Next, the subject was connected to a commercial computer-controlled piston-ventilator (flexiVent, SCIREQ Inc., Montreal, Qc, Canada) for mechanical ventilation and PFTs [11]. A paralytic agent, pancuronium bromide (0.8 mg/ml in saline, Sigma P1918), was then administered to the anesthetized mouse *via* intraperitoneal injection at a volume of 10 ul/gram of body weight to prevent any interference from the subject during the PFTs. Following a brief equilibration period under default mechanical ventilation settings (150 breaths/min, tidal volume of 10 ml/kg, and a PEEP of 3 cmH_2_O), two recruitment maneuvers (inflation to a standard pressure of 30 cmH_2_O over a 3 second and holding for an additional 3 seconds) were performed to open closed lung areas and standardize lung volume history.

A pre-defined script for testing fast acting bronchoprotective therapeutic compounds during a bronchoconstrictive event was initiated (Figure 1B). It started with two deep inflations preceding a succession of two closely spaced nebulizations performed using two separate but identical nebulizer heads (Aeroneb Lab Nebulizer; fine mist model, synchronized with inspiration, operated at 50% duty cycle for 12 s). These were used to deliver two distinct reagents immediately before an automated sequence of closely spaced respiratory mechanics measurements using the forced oscillation technique (Figure 1C).

In humans, exposure to nebulizer compounds is used to assess airway hyper-responsiveness and asthma. Lung function can be assessed before and after subjects are exposed to bronchodilators, bronchoconstrictors, or other agents such as allergens. This allows us to determine the reversibility of bronchial obstruction or the relative airway hyper responsiveness, both of which can be important markers of human airway disease. In our mouse model, we use a similar approach. Albuterol is used as a bronchodilator to prevent the bronchoconstriction seen during methacholine exposure. Investigational compounds can be substituted for either bronchodilator or the bronchoconstrictor to assess their ability to modulate airway responses.

In the present study, all mice received two subsequent saline challenges as this was used as a control for the two nebulizers. They were then divided in two experimental groups: a control (Figure 1, left panel) and an albuterol-treated group (Figure 1**,** right panel), where they received an aerosol of either Albuterol Sulfate or its vehicle (saline) immediately before an aerosolized methacholine-induced bronchoconstrictor challenge. Fifteen seconds after the end of the second aerosol challenge (i.e. saline or methacholine), a loop of closely spaced FOT measurements was initiated. Single frequency (Snapshot-150; 2.5Hz) and broadband (Quick Prime-3; 1–20.5 Hz) FOT measurements were alternated a few seconds apart for a total of 12 measurements per perturbation over a period of approximately 3 minutes. This protocol was repeated a total of four times with increasing concentrations of methacholine (0, 10, 30, 100 mg/ml). The duration of the entire experimental protocol on the machine from initiation of the script to the end is approximately 17 min (4 min 12 sec per cycle × 4 cycles) per mouse. It is important to note that prior to each methacholine challenge, the subjects received a new saline or Albuterol Sulfate aerosol challenge. This was done in order to ensure proper delivery into the airways but also sufficient drug exposure during increasing bronchoconstriction (Figure 1C). In the present study, the same concentration of Albuterol Sulfate (0.083%) was used throughout the entire protocol, similar to what is done in a clinical setting. It was always administered prior to the methacholine challenge in order to prevent nebulization artifacts and delays on the bronchoconstrictor response. The efficacy of this commonly used asthma drug was assessed by comparing peak responses between subject groups for each respiratory mechanic’s parameter studied.

## Results

**Figure 2:**
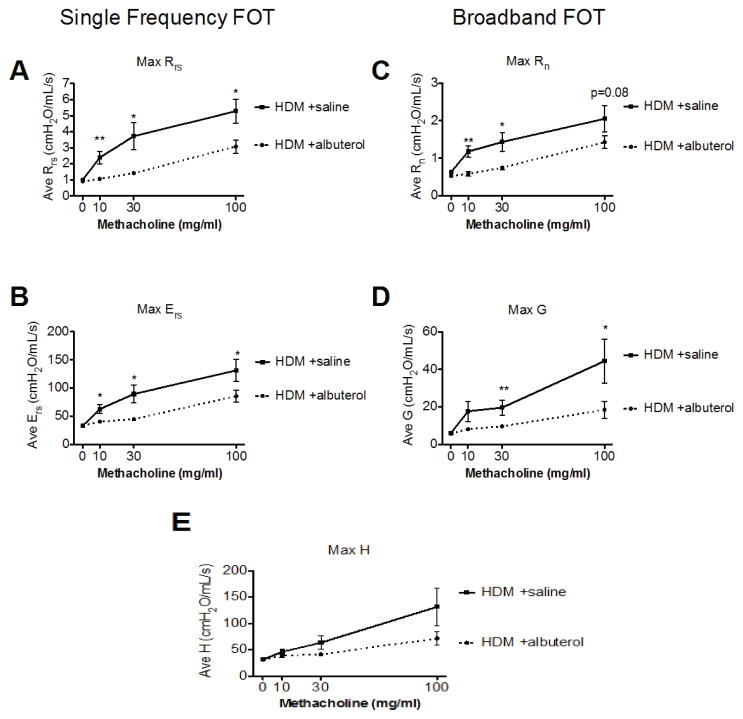
Respiratory mechanics parameters from HDM-sensitized and challenged mice that were either given vehicle (saline-solid line) or albuterol (0.083%–dotted line) immediately before each methacholine challenges. Results are shown as mean ± SEM with n=11–15 mice per group. Statistical significance is shown for each parameter as a comparison between albuterol and saline at the respective challenge. ^*^p<0.05, ^**^p<0.01.

The resistance of the respiratory system (Rrs) reflects the overall airflow resistance at the breathing frequency. Rrs is calculated from the classic single compartment model of the lung being fit to the single frequency FOT experimental data. It includes a contribution from the conducting and peripheral airways, the tissue, and the chest walls. In the present validation study, all mice given albuterol had significantly lower Rrs values compared to those in the control group at all methacholine concentrations (Figure 2A).

The respiratory system elastance (Ers) quantifies the overall stiffness of the entire respiratory system during tidal breathing. It is also calculated from the classic single compartment model of the lung being fit to the single frequency FOT experimental data.

As seen in control mice, Ers typically increases during a bronchoconstrictive event. Under our experimental conditions, the albuterol treatment significantly prevented the methacholine-induced increased in Ers in allergic mice (Figure 2B).

The Newtonian resistance (Rn) is dominated by the resistance of the large conducting airways not involved in gas exchange. This parameter is derived from the Constant Phase Model [24] being fit to the impedance data of broadband FOT measurements [10]. Albuterol treated mice had significantly lower Rn values at the 10 and 30 mg/ml methacholine challenges than their vehicle treated control group. There was also a trend (p=0.08) towards protection at the highest methacholine concentration (Figure 2C).

Tissue damping (G) is also a Constant Phase Model [24] parameter and reflects a measure of the amount of energy that is lost within the tissues as a result of friction. It is closely related to tissue resistance which includes resistance to air flow in the peripheral airways. Following increasing methacholine challenges, there was an increase in the parameter G in the control mice which was significantly blunted in the albuterol treated mice during the 30 and 100 mg/ml methacholine challenges (Figure 2D).

Tissue elastance (H) is an index of tissue stiffness. It is calculated from the Constant Phase Model [24] and represents the ability of the tissues to retract to its original shape. Albuterol treated mice had attenuated measures of H compared to control mice, although the difference between the two experimental groups was not statistically significant at any of the methacholine challenges studied (Figure 2E). Overall, our results for airway physiology parameters measured by FOT are similar to those already published for HDM challenged mice without drug intervention [25].

## Discussion

While we recognize that mouse allergen challenge models are frequently used as a tool for asthma research, we acknowledge that the complexity of the human asthma is unlikely to be adequately modeled in this system. Our goal was to model a specific disease phenotype of asthma, airway hyper-responsiveness, and validate a system to test compounds to attenuate this response in an animal model. As discussed in detail in a review by Kumar and Foster [26], there are several factors observed in mouse models of asthma that are very different from human asthma symptoms. Among the most notable are the different patterns of perivascular and edematous inflammation observed in allergic mouse lungs as compared to human asthmatic lungs, the higher number of eosinophils in mouse lavage (40–80% of total cells in mice versus ~1–5% in lavage from human asthmatics), and the observation of small airway closure independent of smooth muscle contraction [26]. However, given all of these differences, it is widely accepted that bronchoconstrictor challenge results in airway closure and thereby contributes to the mechanism of airway hyper-responsiveness in both human asthmatics and in mouse models of allergic airway disease [27].

Since the most debilitating phenotypes associated with asthma are wheezing and shortness of breath, both of which result from increased bronchoconstriction, fast-acting therapeutic agents are needed for the management of these asthmatic bronchospasms. β-agonists are the most commonly prescribed therapeutic for asthma management but have less efficacy for some subsets of asthmatic patients. With the ever-increasing burden of asthma, new and different targets to alleviate bronchoconstriction are needed as well as the means to screen or test them at the preclinical level. Fast acting drugs, such as albuterol, or novel therapeutic treatments for asthma have been evaluated before in animal studies, however at least in mice, these compounds were never given in the midst of a bronchoconstrictive event. We provide a validated, automated, *in vivo* experimental approach in which potential fast-acting bronchoprotective drugs can be reproducibly tested to advance the development of novel asthma therapeutics.

## References

[R1] 1Statistics, C.N.C.f.H. Asthma. Feb, 2016.

[2] Peat JK, Woolcock AJ, Cullen K (1987). Rate of decline of lung function in subjects with asthma. Eur J Respir Dis.

[3] Halwani R, Al-Muhsen S, Hamid Q (2010). Airway remodeling in asthma. Curr Opin Pharmacol.

[4] Firszt R, Kraft M (2010). Pharmacotherapy of severe asthma. Curr Opin Pharmacol.

[5] Osborne ML, Pedula KL, O’Hollaren M, Ettinger KM, Stibolt T (2007). Assessing future need for acute care in adult asthmatics: the Profile of Asthma Risk Study: a prospective health maintenance organization-based study. Chest.

[6] Dougherty RH, Fahy JV (2009). Acute exacerbations of asthma: epidemiology, biology and the exacerbation-prone phenotype. Clin Exp Allergy.

[7] Dahl R (2006). Systemic side effects of inhaled corticosteroids in patients with asthma. Respir Med.

[8] Beasley R, Pearce N, Crane J, Burgess C (1999). Beta-agonists: what is the evidence that their use increases the risk of asthma morbidity and mortality?. J Allergy Clin Immunol.

[9] Taylor DR, Drazen JM, Herbison GP, Yandava CN, Hancox RJ (2000). Asthma exacerbations during long term beta agonist use: influence of beta(2) adrenoceptor polymorphism. Thorax.

[10] Bates JH, Irvin CG, Farré R, Hantos Z (2011). Oscillation mechanics of the respiratory system. Compr Physiol.

[11] McGovern TK, Robichaud A, Fereydoonzad L, Schuessler TF, Martin JG (2013). Evaluation of respiratory system mechanics in mice using the forced oscillation technique. J Vis Exp.

[12] Shalaby KH, Gold LG, Schuessler TF, Martin JG, Robichaud A (2010). Combined forced oscillation and forced expiration measurements in mice for the assessment of airway hyperresponsiveness. Respir Res.

[13] Bates JH, Irvin CG (2003). Measuring lung function in mice: the phenotyping uncertainty principle. J Appl Physiol.

[14] Finney PA, Belvisi MG, Donnelly LE, Chuang TT, Mak JC (2000). Albuterol-induced downregulation of Gsalpha accounts for pulmonary beta(2)-adrenoceptor desensitization in vivo. J Clin Invest.

[15] Lin R (2012). Chronic treatment in vivo with beta-adrenoceptor agonists induces dysfunction of airway beta(2)-adrenoceptors and exacerbates lung inflammation in mice. Br J Pharmacol.

[16] Tamaoki J, Tagaya E, Kawatani K, Nakata J, Endo Y (2004). Airway mucosal thickening and bronchial hyperresponsiveness induced by inhaled beta 2-agonist in mice. Chest.

[17] Lundblad LK, Rinaldi LM, Poynter ME, Riesenfeld EP, Wu M (2011). Detrimental effects of albuterol on airway responsiveness requires airway inflammation and is independent of β-receptor affinity in murine models of asthma. Respir Res.

[18] Song W, Wei S, Liu G, Yu Z, Estell K (2011). Postexposure administration of a {beta}2-agonist decreases chlorine-induced airway hyperreactivity in mice. Am J Respir Cell Mol Biol.

[19] North ML, Amatullah H, Khanna N, Urch B, Grasemann H (2011). Augmentation of arginase 1 expression by exposure to air pollution exacerbates the airways hyperresponsiveness in murine models of asthma. Respir Res.

[20] Raffay T, Kc P, Reynolds J, Di Fiore J, MacFarlane P (2014). Repeated β2-adrenergic receptor agonist therapy attenuates the response to rescue bronchodilation in a hyperoxic newborn mouse model. Neonatology.

[21] Teixeira VP, Cervilha DA, Cabral LD, Oliveira LM, Incerpi EK (2016). Postnatal overnutrition in mice leads to impaired pulmonary mechanics in response to salbutamol. J Physiol Sci.

[22] Truchetti G, Troncy E, Robichaud A, Gold L, Schuessler T (2014). Respiratory mechanics: comparison of Beagle dogs, Göttingen minipigs and Cynomolgus monkeys. J Pharmacol Toxicol Methods.

[23] Hartney JM, Robichaud A (2013). Assessment of airway hyperresponsiveness in mouse models of allergic lung disease using detailed measurements of respiratory mechanics. Methods Mol Biol.

[24] Hantos Z, Daróczy B, Suki B, Nagy S, Fredberg JJ (1992). Input impedance and peripheral inhomogeneity of dog lungs. J Appl Physiol.

[25] Piyadasa H, Altieri A, Basu S, Schwartz J, Halayko AJ (2016). Biosignature for airway inflammation in a house dust mite-challenged murine model of allergic asthma. Biol Open.

[26] Kumar RK, Foster PS (2012). Are mouse models of asthma appropriate for investigating the pathogenesis of airway hyper-responsiveness?. Front Physiol.

[27] Berend N, Salome CM, King GG (2008). Mechanisms of airway hyperresponsiveness in asthma. Respirology.

